# Dietary Supplements for Erectile Dysfunction: Analysis of Marketed Products, Systematic Review, Meta-Analysis and Rational Use

**DOI:** 10.3390/nu15173677

**Published:** 2023-08-22

**Authors:** Gabriel Cosmin Petre, Francesco Francini-Pesenti, Amerigo Vitagliano, Giuseppe Grande, Alberto Ferlin, Andrea Garolla

**Affiliations:** 1 Unit of Andrology and Reproductive Medicine & Centre for Male Gamete Cryopreservation, Department of Medicine, University of Padova, 35100 Padova, Italy; gabriel.petre@rocketmail.com (G.C.P.); grandegius@gmail.com (G.G.); alberto.ferlin@unipd.it (A.F.); 2 Department of Medicine, Clinical Nutrition Unit, University of Padova, 35128 Padova, Italy; francesco.francini@aopd.veneto.it; 3 Unit of Obstetrics and Gynecology, Department of Biomedical and Human Oncologic Science, University of Bari, 70121 Bari, Italy; amerigo.vitagliano@gmail.com

**Keywords:** erectile dysfunction, dietary supplements, sexual disorder, botanical extract, nutraceuticals, IIEF

## Abstract

The use of nutraceutical products to enhance male sexual performance has a long history, especially with regard to the treatment of erectile dysfunction (ED). Alternative treatments for ED are becoming increasingly popular, with growing interest from consumers, as well as increased revenue for manufacturers. Dietary supplements (DSs), which are a mixture of active ingredients, are mainly sold online. In randomized controlled trials, the molecules contained in DSs have demonstrated varying degrees of effectiveness, or even have no evidence to support their use. However, none of the studies carried out provided sufficient evidence to consider these products a first-line therapy. Therefore, the combination of the various active ingredients, especially in relation to the daily dose, leaves doubts about the real effectiveness. In order to evaluate the potential efficacy of DS formulations, we analyzed the products marketed in Italy using a scoring approach. A systematic review of the literature was performed to evaluate the effect of DS and to detect the active ingredients able to improve erectile function—called effective ingredients (EIs)—and their minimal effective daily dose (mED). A metanalysis identified some nutraceuticals, such as Panax ginseng, Tribulus terrestris and L-arginine, that are able to improve male sexual function. Based on the scoring system, 2 (8%) supplements matched with the cluster of higher expected efficacy, 3 (12%) with the lower efficacy cluster and 20 (80%) matched with the criterion of no expected efficacy. DSs marketed in Italy are usually blends of many substances that are frequently employed at a negligible dose or without any evidence.

## 1. Introduction

Erectile dysfunction (ED) is defined as the inability to obtain and keep a penile erection firm enough for satisfactory sexual performance. This medical condition affects 1 to 10% of adults aged <40 years, and about 30% to 50% of men aged 40–70 years; aside from premature ejaculation, ED represents the most common typology of male sexual disorders [1]. Unsatisfactory sexual performance can cause stress, affect self-confidence and contribute to relationship problems, which impact men’s quality of life (QoL) [2].

The origin of ED can be psychogenic or organic. In about 20% of cases, psychological disturbances are the cause of the disease, while more than 80% of the erectile disorders are organic, both vasculogenic and iatrogenic [3]. It is noteworthy that having trouble obtaining or keeping a satisfying erection can also be a signal of underlying health conditions that need a more in-depth investigation, such as endocrine and non-communicable diseases [4]. Modifiable risk factors, such as overweight/obesity, insulin resistance, diabetes, atherosclerosis and hypertension, together with harmful lifestyles, including cigarette smoking, substance abuse and/or doping drugs, excessive alcohol intake and a sedentary lifestyle, represent the main risk factors for ED [5].

The current medical interventions used to treat ED include oral drugs with phosphodiesterase type 5 (PDE5) inhibitors (representing a first-line treatment); intra-penile prostaglandin administration (intra-urethral applications and intra-cavernous injections); and more recently, low-intensity extracorporeal shockwave therapy [6]. These therapies have been included in the 2023 guidelines of the European Association of Urology (EAU) Sexual and Reproductive Health to manage male sexual dysfunction [7].

The use of botanical extracts or other nutraceutical products to enhance male sexual performance has a long history. Moreover, Ayurvedic and other alternative medicines around the world have always used herbs to help improve symptoms related to ED. Nowadays, a large number of dietary supplements (DSs) formulated with variable ingredients and concentrations are available on the market, highly publicized by the media and widely self-purchased by men with ED. This ease of access could create some concerns about the dosage of the intake of the various active ingredients, the quality of the raw materials of the supplements, possible drug interactions and the real effectiveness of the commercial formulations of the various products [8,9].

Currently, the scientific literature is lacking regarding good-quality studies that can give clinicians clear indications on the efficacy of various active ingredients and which dosages are useful. On this basis, many professionals are dubious about the effectiveness of commercial DSs [10]. Despite there not being a strong recommendation to use DS for ED, patients are increasingly turning to alternative therapies in the hope of reducing side effects and improving their sexual performance without asking for medical help. Moreover, patients are usually embarrassed about their erectile problems, and even in the cases where they would like to consult treating physicians about their sexual health, medical professionals are not willing to address this health problem [11]. Therefore, it has certainly become important to ensure the safety and efficacy of the DSs that are sold so easily worldwide [12].

Taking into consideration only RCTs available in the literature regarding the single active ingredients that were shown to be useful in the treatment of ED, we critically evaluated the efficacy and safety of the DS formulations marketed in Italy as support for the treatment of erectile disorders.

## 2. Materials and Methods

A systematic review of the literature was performed in order to evaluate the clinical efficacy of herbal phytochemicals and other nutrients used to prepare DSs for the management of ED. We searched five databases (Google Scholar, MEDLINE, Scopus, EMBASE and Cochrane Library) to identify randomized controlled trials (RCTs) published in the past decade in this field; the quality was assessed and the risk of bias was estimated using the Preferred Reporting Items for Systematic Reviews and Meta-Analysis (PRISMA) statement and the Cochrane risk of bias tool, respectively. To ascertain the certainty of evidence, the Grading of Recommendations, Assessment, Development and Evaluations (GRADE) framework was used. There was no language restriction applied. The key terms used for the search were male sexual dysfunction, erectile dysfunction, impotence, phytochemical, botanicals, herbal extract, nutraceutical, dietary supplement and traditional medicine, in combination with the International Index of Erectile Function (IIEF).

To be eligible for this systematic review and metanalysis, we considered studies with the following characteristics: (a) with the aim to rule out the possible interactions between substances, only RCTs that used one drug were enrolled, excluding any kind of pharmacological treatment; (b) any etiology of ED (e.g., psychogenic, vascular and drug induced) and severity (mild, moderate and severe); and (c) studies that assessed erectile function through validated methods, such as IIEF-15, IIEF-5, IIEF-6, IIEF-EF, questionnaire on male erectile dysfunction (KEED), O’Leary’s questionnaire and the Derogatis Interview for Sexual Function (DISF), Erectile Hardness Scale (EHS) and Flo-Mediated Dilatation (FMD), while excluding “self-reported patient satisfaction” since it is not quantifiable numerically.

### 2.1. Data Analysis

A meta-analysis was conducted only if two or more RCTs were undertaken for the same molecule and outcomes were available. Statistical analysis was performed independently by two authors (A.V., G.P.) using Review Manager Version 5.4 (The Cochrane Collaboration, Software Update, Oxford, London). The results were compared and any difference was resolved via discussion with a third author (A.G.). Continuous variables were compared by using the means and standard deviations of changes from the baseline outcomes. All analyses were carried out with an intention-to-treat approach (mean changes per randomized man). The results were expressed as mean differences (MDs) between groups (95% CI). For the mean difference approach, the standard deviations were used together with the sample sizes to compute the weight given to each study. If mean changes from the baseline measurements were not reported, they were calculated as differences between the final and baseline means (μd = μ1 − μ2). Changes in standard deviations were calculated by using the formula Sd change = sqrt (SD12 + SD22 − (2 × corr × SD1 × SD2)), where the correlation coefficient was calculated as corr = (SD12 + SD22 − SD change2)/(2 × SD1 × SD2). The significance level was set at *p* < 0.05. Heterogeneity was assessed using the Higgins I^2^. The results are displayed graphically using forest plots.

### 2.2. Risk of Bias

Two authors (G.P., A.V.) independently assessed the methodological quality of the included studies by using the Cochrane Collaboration’s tool for bias risk assessment (Higgins JPT, Green S. Cochrane Handbook for Systematic Reviews of Interventions Version 5.1.0 (updated March 2011); The Cochrane Collaboration, 2011; available from http://handbook.cochrane.org (accessed on 1 May 2023). The following domains were assessed: random sequence generation, allocation concealment, blinding of participants and personnel, blinding of outcome assessors, incomplete outcomes, selective data reporting and other sources of bias. The authors’ judgments were expressed as “low risk”, “high risk” or “unclear risk” of bias for each domain. The authors’ scores were compared and disagreements were resolved via consensus.

### 2.3. Definition of Potential Active Ingredients, Minimal Effective Dose and Evaluation of Commercial DS Formulation

The selected articles allowed us to clearly identify effective ingredients (EIs), namely, the substances proposed as having a clinically demonstrated efficacy on the improvement of erectile function. To establish the likely efficacy of each EI, we considered only those having at least one RCT demonstrating a positive, negative or no effect on the erectile function. We considered the lowest effective dose reported in RCTs for each EI as the minimal effective daily dose (mED) able to improve ED.

In order to discover the commercial formulations of DS available on the web market sold with the purpose of improving erectile function, we employed the Google search engine using the following terms: “male supplements” and “supplements for erectile dysfunction”; this identified a lot of online retailers (such as Amazon, eBay, pharmacies, para-pharmacies and various supplement shops) who sold various DSs with an indication to improve ED. Moreover, to be sure that all the supplements found were regularly saleable in Italy and possibly find others to be able to analyze, we referred to the register of dietary supplements available on the website of the Italian Ministry of Health [13], updated to 1 May 2023, that contained the list of all products notified in this country. From this list, we extrapolated all the supplements regularly sold in Italy with the indication of supporting erectile function.

In recent systematic reviews by our group on DS formulation for male and female infertility [14,15], we suggested a formula to evaluate the expected efficacy of a DS based on the whole composition, specifically, each formulation was evaluated in relation to the fact that if each single active ingredient was considered an EI, and whether each one reached at least the mED based on the suggested daily dose of intake. The equation was evaluated using an adapted version of the validated scoring system that The American Heart Association has developed for analyzing scientific evidence from clinical trials [16].

The scoring system assigned a grade (A, B, C or D) to each EI based on the level of published evidence that they demonstrate. An ingredient was designated evidence level A if it demonstrated a net positive impact in multiple RCTs. Level B was assigned if a net positive impact was demonstrated in at least 1 RCT. Level C was assigned if multiple RCTs showed opposing results for the same outcome, and level D was given for ingredients showing negative or no effect on the analyzed outcomes. Regarding commercial formulations of DSs, each EI was scored using the scoring system on the basis of the previously described method and whether it was present in the supplements in an amount that reached at least the minimal effective daily dose (Table 1).

Once a category was designated to each ingredient, a score was assigned as follows: A = 5, B = 3, C = 1, D = −1. Subsequently, the scores were designated to each of the supplements depending on their respective compositions: briefly, the score of each ingredient constituting the supplement (i.e., A = 5, B = 3, C = 1, D = −1) was summed. Then this score was weighted for the total number of ingredients in the supplement (N). Finally, in order to reward those supplements with only class A and B ingredients, the relative score was multiplied by the number of class A ingredients plus half the number of class B ingredients, resulting in the final score of the supplement:

Score=(5A+3B+C−D)N×(1+A+B2)

Given the distribution of the scores resulted in three main clusters, we classified the DSs into three categories, resembling the efficacy of the ingredients: higher expected efficacy (corrected score ≥ 2), lower expected efficacy (≥1 corrected score < 2) and no expected efficacy (corrected score < 1).

## 3. Results

**Figure 1:**
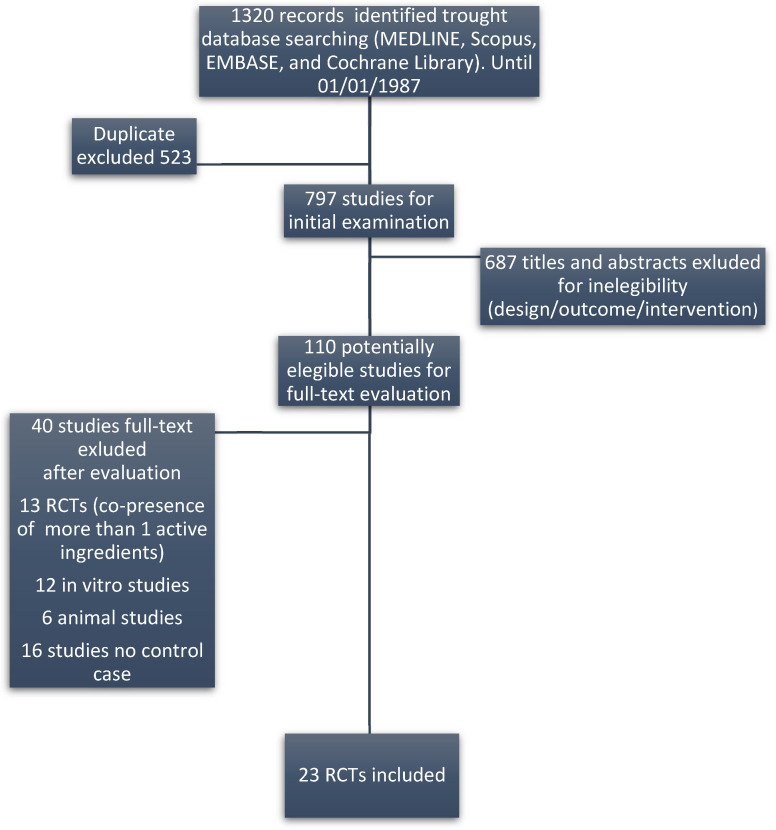
Flow diagram of the eligible papers.

The literature review on ingredients identified 194 records published from 1987 onward, yielding 110 potentially eligible studies. Of these, 87 were excluded according to the reasons reported in Figure 1. A total of 23 studies (only RCTs) were finally included in this study. Most of the studies reviewed included patients with various causes of ED.

A total of 41 different active ingredients were used by manufacturers for the various formulations of DSs. Based on the literature analysis, we found that 33 out of the 41 ingredients had no reported efficacy on ED. The list of the eight ingredients with recognized clinical evidence of efficacy, respective references, associated clinical outcome, mED and employed daily doses are summarized in Table 2. In detail, six EIs with evidence of efficacy were supported by at least two RCTs, namely, Eurycoma longifolia, Panax ginseng, L-arginine, Corynanthe yohimbe, Tribulus terrestris and Pinus pinaster. The remaining two EIs, namely, Crocus sativus and Withania somnifera, had only one positive reference. It is noteworthy that in the European Union, yohimbine cannot be sold as a supplement based on the European Food Safety Authority’s (EFSA’s) scientific opinion [17]. Therefore, two DS, that were found online containing C. yohimbe extract were not considered in the evaluation of the expected efficacy of the various commercial formulations. The characteristics of the 27 DSs marketed in Italy are summarized in Table 3, reporting the respective composition, recommended daily dose, grade of evidence for each ingredient and efficacy scores.

All the supplements contained mixtures of EIs ranging from 2 to 13 substances, with an average number of more than 5 ingredients. It was remarkable that all 27 supplements contained at least one ingredient without any evidence of efficacy. In 24 formulations (88.8%), there were ingredients with a dose below the mED. In particular, 3 supplements (DS 4, 5 and 7) contained 12 ingredients, of which 9 lacked demonstrated efficacy. DS 4 and 7 contained at least three ingredients dosed below the mED and only one formulation (DS 5) had an EI at the right dose.

Among the DSs, the most used active ingredient was zinc, even if it had no supporting literature in this field. Among the EIs, L-arginine was the most used ingredient, followed by Tribulus terrestris and Panax ginseng. These three ingredients were used in more than 90% of the formulations, whereas each of the remaining EIs was found in less than 5% of the products. Indeed, E. longifolia and W. somnifera, which had literature supporting the improvement of functional hypogonadism or late-onset hypogonadism (LOH), were poorly taken into consideration by manufacturers.

**Figure 2:**
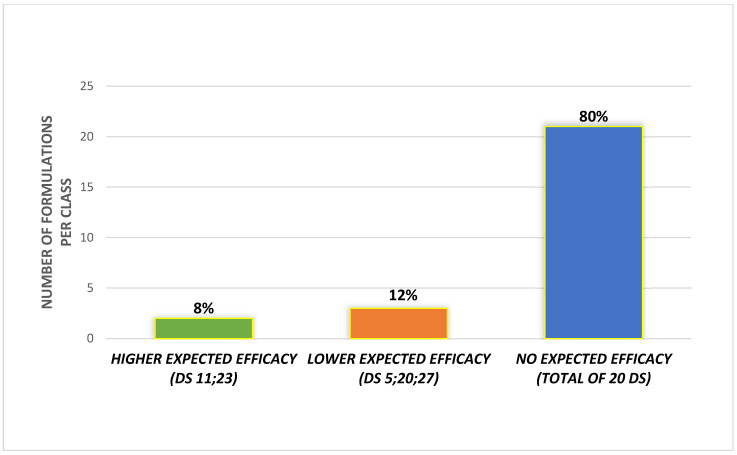
Distribution of supplements among the classes of expected efficacy.

Regarding the scoring approach, we considered only the 25 out of 27 DS that did not contain C. yobimbe or its alkaloids. Based on the scoring system, 2 (8%) supplements matched with the cluster of higher expected efficacy, 3 (12%) with the lower expected efficacy group and 20 (80%) matched with the criterion of no expected efficacy (Figure 2).

### Meta-Analysis

**Figure 3:**
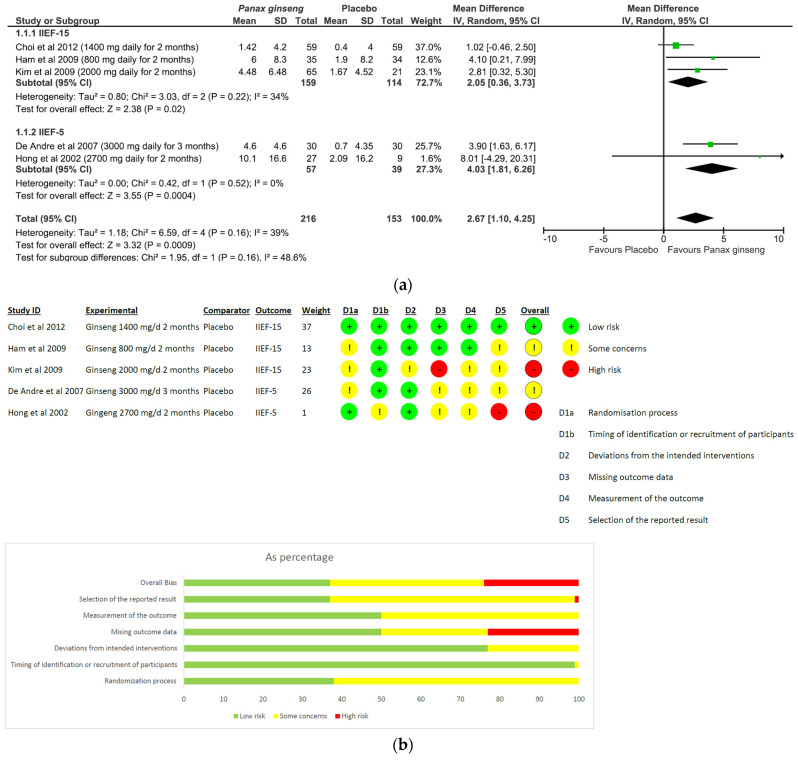
*P. ginseng* plots: (**a**) meta-analysis results; (**b**) risk of bias [20,21,22,23,24].

**Figure 4:**
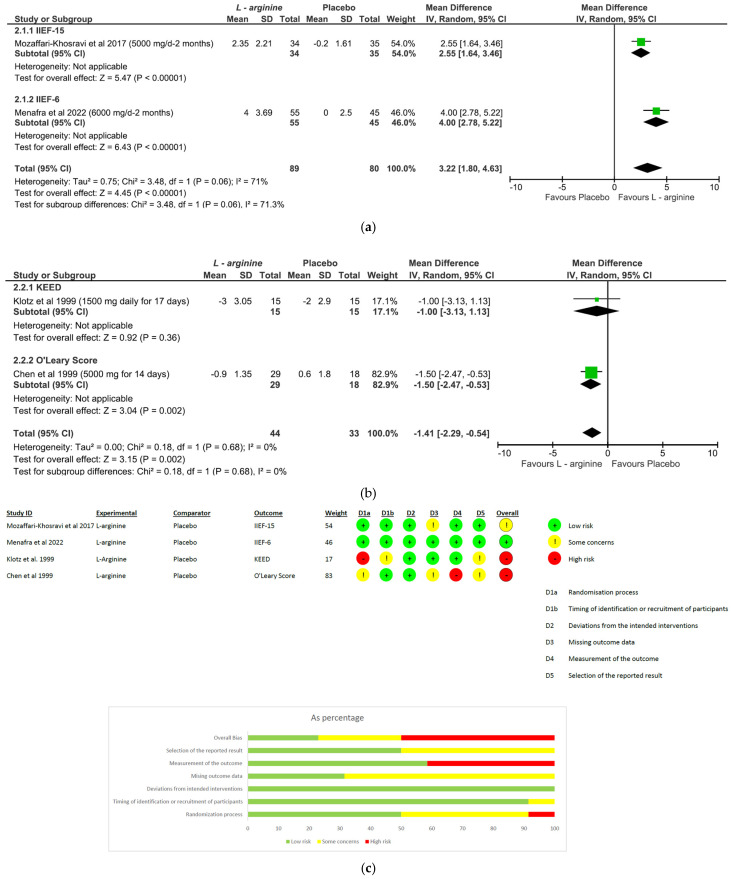
*L-arginine* plots: (**a**,**b**) meta-analysis results; (**c**) risk of bias [25,26,27,28].

**Figure 5:**
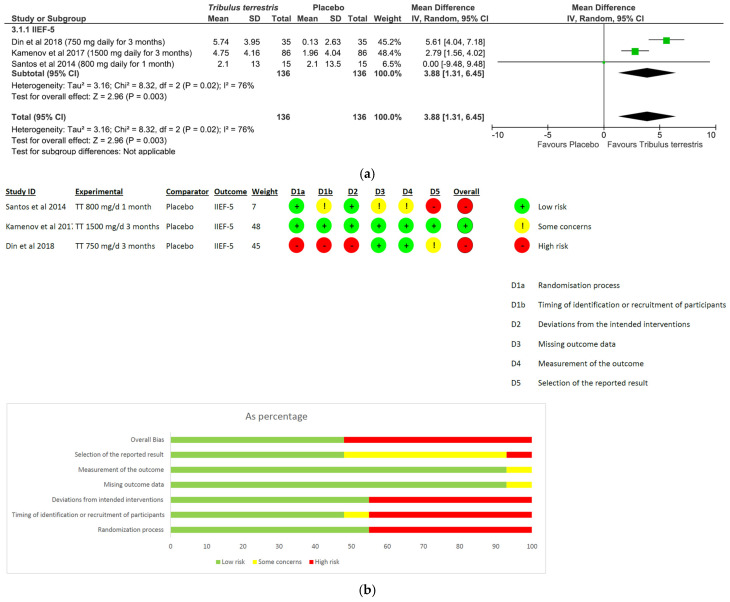
*T. terrestris* plots: (**a**) meta-analysis results; (**b**) risk of bias [33,34,35].

**Figure 6:**
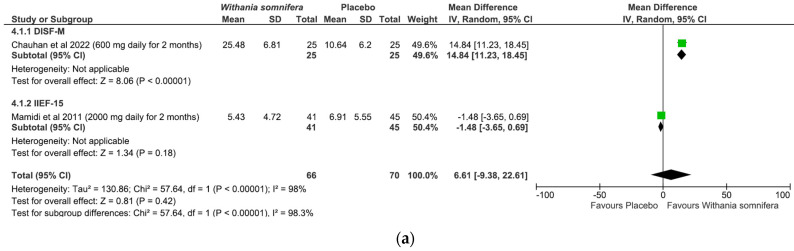

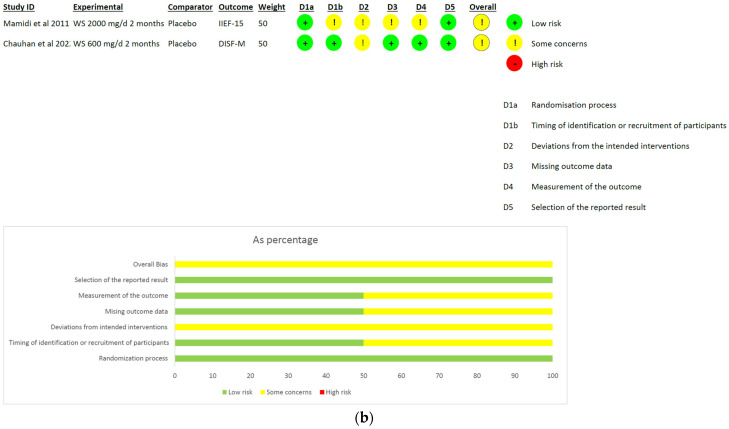
*W. somnifera* plots: (**a**) meta-analysis results; (**b**) risk of bias [37,38].

A meta-analysis was feasible only for the following EIs: P. ginseng, L-arginine, T. terrestis and W. somnifera. Fourteen RCTs were suitable for the meta-analysis. RCTs using Corynanthe yohimbe were not included.

(I) *Panax ginseng*: A total of five studies and 369 patients were included in the analysis (intervention group N = 216 patients; control group N = 153 patients). The intervention was associated with a significant improvement in erectile performance as assessed using IIEF-15 and IIEF-5 (pooled MD = 2.67, [95% CI 1.10, 4.25], *p* = 0.0009, I^2^ = 39%) (Figure 3a). The overall risk of bias was about 35% high, 40% with some concerns and the remaining percentage was low (Figure 3b).
(II) *L-arginine:* The analysis included a total of four studies and 246 patients (intervention group N = 133 patients; control group N = 113 patients). The intervention was associated with a significant improvement in erectile performance as assessed using IIEF-15 and IIEF-6 (pooled MD = 3.22, [95% CI 1.80, 4.63], *p* < 0.00001, I^2^ = 71%; Figure 4a), as well as the KEED and O’Leary scores (pooled MD = −1.41, [95% CI −2.29, −0.54], *p* = 0.002, I^2^ = 0%; Figure 4b). The overall risk of bias was 50% high, about 37% unclear and low for the remaining percentage (Figure 4c).
(III) *Tribulus terrestris:* A total of three studies and 272 patients were included in the analysis (intervention group N = 136 patients; control group N = 136 patients). The intervention was associated with a significant improvement in erectile performance as assessed using IIEF-5 (pooled MD = 3.88, [95% CI 1.31, 6.45], *p* = 0.003, I^2^ = 76%; Figure 5a). The overall risk of bias was 50% high and 50% low (Figure 5b).
(IV) *Withania somnifera:* The analysis included a total of two studies and 136 patients (intervention group N = 66 patients; control group N = 70 patients). No difference was found between the comparators when pooling data about erectile performance as assessed with both DISF-M and IIEF-15 (pooled MD = 6.61, [95% CI −9.38, 22.61], *p* = 0.42, I^2^ = 98%; Figure 6a). Notably, a significant difference between subgroups was found (*p* < 0.00001), highlighting a beneficial effect of the intervention in terms of DISF-M (*p* < 0.00001) but not IIEF-15 (*p* = 0.18). Furthermore, in the case of Mamidi et al. (IIEF-15), there were patients suffering from psychogenic ED, and therefore, at an organic level, there was no disorder, which was maybe why the active ingredient did not have a positive response. The overall risk of bias was unclear, especially in relation to the study by Mamidi et al., which presented four out of six participants who were uncertain (Figure 6b).

## 4. Discussion

Herbal extracts have been used by various traditional medicine systems (Chinese, Indian, etc.) for the treatment of ED and the improvement of sexual health. Although the used plants have a long popular tradition, very often there is a lack of scientific data in relation to their efficacy [41]. A large number of DSs containing different ingredients have been proposed for the treatment of male sexual dysfunction. Despite these products having a huge market, few well-designed studies have been conceived to evaluate their efficacy [42].

To the best of our knowledge, this study represents the first critical analysis of DS formulations marketed for ED. Notably, all evaluated DSs contained one or more ingredients with no evidence of efficacy supported by the literature. Moreover, many ingredients whose efficacy was demonstrated by clinical trials were dosed under the mED, raising several doubts about their real impact on sexual function.

In the present study, we observed that the most employed EI was L-arginine, which was contained in about 63% of DS formulations, but always used at an incorrect dosage (the mED is 5 g). The physiology underlying penile erection is known to be a dynamic vascular process involving the relaxation of arterial and trabecular smooth muscle in the corpus cavernosum [43]. Nitric oxide (NO) is considered to be the primary mediator of a penile erection and derives from two different sources. First of all, NO is produced by the neuronal enzyme NO synthase (NOS) (non-cholinergic nerve axons) of the penis and by endothelial NOS (eNOS) from endothelial cells of the corpora cavernosa [44]. The release of NO from neuronal NOS activates eNOS NO production, which subsequently binds (in quantitatively much higher amounts) to the enzyme guanylate-cyclase in vascular smooth muscle cells to generate a second messenger, namely, cyclic guanosine monophosphate (cGMP). At this point, cGMP inside muscle cells causes relaxation and vasodilation, resulting in penile erection. The amino acid L-arginine is the only substrate used by NOS [45]. To improve the activity of eNOS, pycnogenol (which is an extract of the bark of Pinus pinaster, which consists of a concentrate of polyphenols, mainly procyanidins) is also used together with arginine. Pycnogenol improves ED symptoms by activating eNOS, which, in turn, increases NO production and promotes vasodilation in the presence of copious amounts of arginine [46]. In light of the final positive result of the metanalysis (pooled MD = 3.22, [95% CI 1.80, 4.63], *p* < 0.00001, I^2^ = 71%) and the generally low risk of bias, it can be stated that L-arginine could be useful in the treatment of organic ED.

Tribulus terrestris was found to be the second most used ingredient in the evaluated DSs. Steroidal saponins, such as protodioscin, furostanol, neotigogenin, tigogenin, gitogenin, neogitogenin and diosgenin, are considered to be the main active component of T. Terrestris. The saponins fraction was suggested to positively influence testosterone production and improve libido and erectile function [47]. In fact, T. terrestris was shown to support hormonal function via the conversion of protodioscine to dehydroepiandrosterone (DEHA), which is the base molecule for the synthesis of testosterone [48]. Furthermore, diosgenin exerts a well-known protective effect on micro-circulation, and therefore, chronically using Tribulus could improve erectile function [49]. Based on the result of the present meta-analysis (pooled MD = 3.88, [95% CI 1.31, 6.45], *p* = 0.003, I^2^ = 76%), we stated that T. terrestris could be useful in supporting organic ED. However, there are general doubts regarding its real effectiveness due to the high average risk of bias relating to the RCTs analyzed, as well as the high heterogeneity between them.

Panax ginseng, also known as Korean ginseng, was present in about 52% of the supplements. Preparations based on ginseng extracts exert antioxidant, antidiabetic and immunomodulatory activity, and have aphrodisiac properties [50]. All RCTs analyzed in this meta-analysis showed that ginseng was effective in the treatment of erectile dysfunction. Efficacy was observed for doses ranging from 0,8 to 3 g dry extract/day. In this meta-analysis, we included five studies with a high or not fully understood risk of bias (except the paper by de Andre et al.), limiting the weight of the evidence. Ginsenosides are a class of tri-terpenoid saponins with a steroid structure, which is detectable only in plants of the genus Panax. They are the active ingredients to which the effectiveness of ginseng is attributable [51]. Physiological data highlight the ability of ginsenosides to increase the penis blood flow by improving the activity of eNOS. Some animal models showed a dose-dependent relationship between ginseng and NO production in the corpora cavernosa [52].

Therefore, based on the following result of the meta-analysis (*pooled MD = 2.67*, [95% CI 1.10, 4.25], *p* = 0.0009, I^2^ = 39%), we can state that P. ginseng is an herbal extract that is useful in the treatment of organic ED. It should be mentioned that the analyzed RCTs generally presented some concerns regarding the general risk of bias. However, the herbal extract is to be considered useful because all the RCTs in which it was tested showed positive effects.

W. somnifera was included in a single nutraceutical product. Also known as Ashwagandha, it is an adaptogen, particularly its root powder, and has been used for centuries in Indian medicine. It contains a wide range of different classes of chemical constituents, such as alkaloids, triterpene lactones and flavonoids. The main phytochemical constituent is withaferin A, in association with other withanolides [53]. In vitro and in vivo studies demonstrated the plant to be anti-inflammatory, neuroprotective, antitumor, antimicrobial, antistress, antidiabetic and cardioprotective [54]. Lopresti et al. showed that a daily intake of 240 mg of Ashwagandha dry extract results in a significant reduction in Hamilton anxiety rating scale (HAM-A) and stress scale-21 (DASS-21) values, and was associated with a reduction in morning cortisol and a consequent improvement in the testosterone–cortisol ratio [55]. Moreover, in some RCTs, ashwagandha administration showed a positive effect along the hypothalamic–pituitary–gonadal axis [56,57]. In oligozoospermic patients, Ambiye et al. demonstrated an increased LH level from 3.97 to 5.31 mIU/mL after three months of administration of 2100 g/d ashwagandha dry extracts [58]. Again, physiologically diminished LH and testosterone levels were observed in hypogonadal men suffering from ED, which were completely restored after treatment with W. somnifera [59]. The final result of the metanalysis was not in favor of the use of W. somnifera in the treatment of organic ED. However, it must be stated that there was a very high heterogeneity between the two analyzed studies, and the negative study presented a high risk of bias. Therefore, we cannot conclude that W. somnifera is not useful in the treatment of organic ED.

Finally, it is also important to underline that unverified ingredients may be present in some DSs. Tucker et al. reported that Food and Drug Administration’s Tainted Supplements database resulted in a total of 776 adulterated dietary supplement entries from 2007 to 2016. In the majority of cases (757 of 776 (97.6%)), supplements contained ingredients that were not declared on the label. Among supplements for sexual enhancement, nearly half were adulterated and all adulterated formulations were found to contain unapproved drug ingredients [60].

The main points of this work were as follows: (I) For some EIs, such as Eurycoma and C. sativus, it was not possible to perform the meta-analysis. (II) Although C. yohimbe has a positive effect on erectile function, it was excluded from the final DS analysis in accordance with the EFSA’s scientific opinion; in Europe, it cannot be used as a food supplement because it increases cardiovascular risk. Moreover, given that the four RCTs were too heterogeneous, a meta-analysis was not performed. (III) DS formulations available on the Italian market (most likely in Europe) are of poor functional quality and are mixtures of many ingredients without support from the literature. (IV) EIs, when present, are almost always underdosed.

The strength of this work is related to the fact that the meta-analysis allowed us to clarify that some herbal extracts and nutrients, such as Ginseng, Tribulus and Arginine, are able to improve male sexual function. Even if the included studies are not always of good quality, the number of analyzed patients was relevant and the choice to include only evidence from selected RCTs minimized the biases. Instead, the main limitation of this work was that for each EI, there were some risks of bias regarding the methodological quality of the various RCTs, and this could have affected the conclusion drawn. In fact, we can only state that the various EIs are potentially effective in improving erectile function on average. Furthermore, we can hypothesize that some dietary supplements contain unverified ingredients, thus altering the real effect of marketed products.

In conclusion, to be effective, a DS should be well-designed to act both acutely on the production of nitric oxide (locally in the penis) and chronically by normalizing the hormonal framework. Based on the current scientific literature, our study may assist clinicians in choosing the DS formulations that are most likely to be effective in relation to erectile disorders, thus providing the best supplementation for each patient. Furthermore, our work could be useful for designing formulations of good-quality and evidence-based DSs. Moreover, other RCTs on the various EIs are needed, improving the number of included patients and the inclusion criteria, in order to have more solid evidence on single molecules.

## Figures and Tables

**Table 1:** EI assigned grades summarized based on RCTs’ evidence. +: positive RCT; −: negative RCT.

	Reached mED	
**RCT Characteristics**	Yes	Not
≥2 + RCTs	A	B
1 + RCT	B	C
≥2+/≤1 − RCT	B	C
≥2+/> 1 − RCT	C	D
No evidence	D	D

**Table 2:** Active ingredients with evidence of efficacy, evaluated outcomes, references, employed daily dose and minimal effective dose (mED).

Active Ingredient	Reference	Participant Characteristics (Number; Age; Type of ED)	Duration of Treatment	Evaluated Outcomes (Baseline Value; End of Treatment vs. Baseline)	Employed Daily Dose	Minimal Effective Dose (mED)
*Eurycoma longifolia*	[18] [19]	**T**: 18; 47.38 ± 5.03; all types **P**: 19; 47.38 ± 5.03; all types **T**: 52; 43.6 ± 6.52; all types **P**: 50; 42.8 ± 6.73; all types	6 months 3 months	**T**: ↑ IIEF-15; 18.66 ± 2.43; ∆2.82 * ± 2.20 (*p* < 0.05) **P**: ↑ IIEF-15; 20.50 ± 2,10; ∆1.00 ± 2.33 **T**: ↑ IIEF-15; 25.36 ± 0.47; ∆1.43 * ± 0.46 (*p* < 0.001) **P**: no significant differences	200 mg 300 mg	200 mg
*Panax ginseng*	[20] [21] [22] [23] [24]	**T**: 35; 53.20 ± 9.70; all types **P**: 34; 50.80 ± 8.00; all types **T**: 59; 57.49 ± 7.94; all types **P**: 59; 57.32 ± 8.41; all types **T**: 65; 57.51 ± 1.24; all types **P**: 21; 57.51 ± 2.02; all types **T**: 27; 54.00; all types **P**: 9; 54.00; all types **T**: 30; 52.60; all types **P**: 30; 54.30; all types	2 months 2 months 2 months 2 months 3 months	**T**: ↑ IIEF-15; 17.20 ± 9.4; ∆6.00 * ± 8.30 (*p* = 0.003) **P**: ↑ IIEF-15; 17.70 ± 8.2; ∆1.90 ± 8.20 **T**: ↑ IIEF-15; 17.17 ± 2.57; ∆1.42 * ± 4.20 (*p* < 0.05) **P**: ↑ IIEF-15; 17.56 ± 2.89; ∆0.40 ± 4.00 **T**: ↑ IIEF-15; 11.89 ± 5.89; ∆4.48 * ± 6.48 (*p* < 0.001) **P**: ↑ IIEF-15; 11.38 ± 4.78; ∆1.67 ± 4.52 **T**: ↑ IIEF-5; 10.60 ± 7.41; ∆10.10 * ± 16.60 (*p* < 0.01) **P**: ↑ IIEF-15; 10,60 ± 7.41; ∆2.09 ± 16.20 **T**: ↑ IIEF-5; 16.40 ± 2.90; ∆4.60 * ± 4.60 (*p* < 0.01) **P**: ↑ IIEF-5; 17.00 ± 3.10; ∆0.70 ± 04.35	800 mg 1400mg 2000 mg 2700 mg 3000 mg	800 mg
*L-arginine*	[25] [26]	**T**: 34; 51.58 ± 2.67; vasculogenic **P**: 35; 51.31 ± 2.65; vasculogenic **T**: 55; 50.00 ± 14; vasculogenic **P**: 45; 53.00 ± 10; vasculogenic	2 months 3 months	**T**: ↑ IIEF-15; 16.32 ± 1.36; ∆2.35 * ± 2.21 (*p* < 0.001) **P**: ↓ IIEF-15; 16,11 ± 1.30; ∆ −0.20 ± 1.61 **T**: ↑ IIEF-6; 20,00 ± 6.30; ∆4.00 * ± 3.69 (*p* < 0.0001) **P**: ↔ IIEF-6; 20,00 ± 5,00; ∆0.00 ± 2.50	5000 mg 6000 mg	6000 mg
	[27] [28]	Cross-over; 15; 51.60; all types **T**: 29; range 55–75; all types **P**: 18; range 55–75; all types	17 days 2 weeks	**T: ↓** KEED; 21.9 ± 2.7; −∆3.0 ± 3.0 **P: ↓** KEED; 21.9 ± 2.7; −∆2.0 ± 2.9 **T**: ↓ O’Leary score; 18.6 ± 1.3; −∆0.9 ± 1.3 **P**: ↑ O’Leary score; 19.2 ± 1.7; ∆0.6 ± 1.8	1500 mg 5000 mg	
*Corynanthe yohimbe*	[29] [30]	Cross-over; 48; 18 to 70; psychogenic **T**: 41; 53.9; all types **P**: 42; 51.3; all types	10 weeks 2 months	↑ NPT in 62% of patients (*p* < 0.05) T: ↑ NPT in 71% of treated (*p* = 0.01) P: ↑ NPT in 45% of patients	18 mg 30 mg	18 mg
	[31] [32]	Cross-over; 100; 56; all types Cross-over; range 25–51; 51; all types	10 weeks 25 days	↑ NPT in 42.6% of patients (*p* = 0.42) ↑ Rigiscan in 44% of patients	18 mg 36 mg	
*Tribulus terrestris*	[33] [34]	**T**: 35; 55.69 ± 9.35; all types **P**: 35; 58.38 ± 9.71; all types **T**: 86; 44.11 ± 12.37; all types **P**: 86; 41.18 ± 12.36; all types	3 months 3 months	**T**: ↑ IIEF-5; 10.71 ± 3.01; ∆5.74 * ± 3.95 (*p* < 0.001) **P**: ↓ IIEF-5; 10.75 ± 3.01; −∆13.00 ± 2.63 **T**: ↑ IIEF-5; 18.01 ± 3.21; ∆4.75 * ± 4.16 (*p* < 0.001) **P**: ↑ IIEF-5; 18.22 ± 3.44; ∆1.96 ± 4.04	750 mg 1500 mg	750 mg
	[35]	**T**: 15; 60 ± 9.40; all types **P**: 15; 63 ± 7.90; all types	1 month	**T**: ↑ IIEF-5; 13.20 ± 13.00; ∆2.10 * ± 13.00 (*p* = 0.0004) **P**: ↑ IIEF-5; 11.60 ± 13.50; ∆2.10 * ± 13.50 (*p* = 0.0004)	800 mg	
*Crocus sativus*	[36]	**T**: 15; 36.60 ± 8.30; SSRi related ED **P**: 15; 40.05 ± 9.40; SSRi related ED	1 month	**T**: ↑ IIEF-15; 20.70 ± 4.30; ∆4.50 * ± 2.50 (*p* < 0.001) **P**: ↓ IIEF-15; 21.20 ± 3.10; ∆−2.50 ± 4.60	30 mg	30 mg
*Withania somnifera*	[37]	**T**: 25; 34.32 ± 3.21; all types **P**:25; 35.20 ± 3.66; all types	2 months	**T**: ↑DISF-M; 62.92 ± 4.75; ∆9.8 * ± 9.80 (*p* < 0.0001) **P**: no significant differences	600 mg	600 mg
	[38]	**T**: 41; 21 to 40; psychogenic **P**: 45; 21 to 40; psychogenic	2 months	**T**: ↑ IIEF-15; 38.80 ± 4.72; ∆5.43 * ± 4.72 (*p* < 0.01) **P**: ↑ IIEF-15; 38.97 ± 5.55; ∆6.91 * ± 5.55 (*p* < 0.01)	2000 mg	
*Pinus pinaster*	[39] [40]	**T**: 21; 46.50 ± 12.5; all types **P**: not declared **T**: 32; 49.00 ± 12.50; vasculogenic **P**: 21; 50.75 ± 8.20; vasculogenic	3 months 4 months	**T**: ↑ IIEF-5; 12.60 ± 1.10; ∆4.20 * ± 0.95 (*p* < 0.019) **P**: ↓ IIEF-5: 11.30 ± 1.30; ∆−2.40 * ± 1.25 (*p* < 0.01) **T**: ↑ IIEF-5; 9,0; ∆1.85 * (*p* < 0.019) **P**: ↓ IIEF-5; 10.8; ∆−1.25	120 mg 120 mg	120 mg

* means that the value is significant; ∆: End of Treatment vs. Baseline; ↑: improved; ↔/↓: no effect; T: treatment group; P: placebo group; IIEF: International Index of Erectile Function Questionnaire; DISF-M: Derogatis Interview for Sexual Function; NTP: Nocturnal Penile Tumescence test; KEED: Cologne Erectile Inventory; O’Leary score: O’Leary’s Sexual Function Inventory.

**Table 3:** List of dietary supplements (DS**s**) with suggested daily dose. S: score of supplement’s expected efficacy; EV: scoring system assigned grade (A, B, C or D) to active ingredients in relation to the literature and achievement of mED. Ingredients without proven efficacy are in italics.

Active Ingredient	DS 1		DS 2		DS 3		DS 4		DS5		DS 6		DS 7	
	S = −0.67		S = −0.50		S = −0.50		S = −0.58		S = 0.50		S = −0.67		S = −0.46	
	Daily Dose	EV	Daily Dose	EV	Daily Dose	EV	Daily Dose	EV	Daily Dose	EV	Daily Dose	EV	Daily Dose	EV
Tribulus terrestris	200 mg	C					200 mg	C	1200 mg	B			2000 mg	B
Crocus sativus														
Panax ginseng			100 mg	B	200 mg	B	200 mg	B	960 mg	A			125 mg	B
L-arginine					100 mg	C	1000 mg	C	600 mg	C	100 mg	C	800 mg	C
Withania somnifera														
Eurycoma longifolia														
Pinus pinaster														
Corynanthe yohimbe														
*L-taurine*	200 mg	D	200 mg	D			300 mg	D	180 mg	D			200 mg	D
*L-carnitine*											100 mg	D		
*L-citrulline*							150 mg	D	210 mg	D	70 mg	D	1000 mg	D
*Muira puama*	100 mg	D	100 mg	D			100 mg	D	600 mg	D				
*Trigonella foenum-graecum*			150 mg	D	100 mg	D					400 mg	D		
*Ginkgo biloba*					200 mg	D								
*Hawthorn berry*					200 mg	D								
*Punica granatum*					30 mg	D								
*Cuscuta chinensis*					20 mg	D								
*Black Pepper*							15 mg	D	225 mg	D				
*Zingiber officinale*													50 mg	D
*Turnera diffusa*														
*Vitamin C*							50 mg	D	30 mg	D			50 mg	D
*Vitamin B6*							9.5 mg	D	5.7 mg	D			2 mg	D
*Vitamin B9*													200 mcg	D
*Vitamin E*	6 mg	D					15 mg	D	9 mg	D			15 mg	D
*Vitamin D*													5 mg	D
*Vitamin B1*														
*Vitamin H*														
*Vitamin B12*														
*Vitamin B3*														
*Magnesium*							100 mg	D	60 mg	D				
*Zinc*	7.5 mg	D	5 mg	D	15 mg	D	15 mg	D	9 mg	D	14 mg	D	12 mg	D
*Selenium*														
*Alpha lipoic acid*														
*Aspartic acid*														
*Bambusa arundinacea*														
*Epimedium acuminatum*														
*Capsicum annuum*														
*Caffeine*														
*Paullinia capuana*														
*Chlorella pyrenoidosa*														
*Lepidium meyenii*	150 mg	D	200 mg	D	200 mg	D	200 mg	D	1200 mg	D	200 mg	D	200 mg	D
**Active Ingredient**	**DS 8**		**DS 9**		**DS 10**		**DS 11**		**DS 12**		**DS 13**		**DS 14**	
	**S = −1**		**S = −0.33**		**S = −0.66**		**S = 2**		**S = −0.67**		**S = −0.78**		**S = −0.38**	
	**Daily Dose**	**EV**	**Daily Dose**	**EV**	**Daily Dose**	**EV**	**Daily Dose**	**EV**	**Daily Dose**	**EV**	**Daily Dose**	**EV**	**Daily Dose**	**EV**
Tribulus terrestris													75 mg	C
Crocus sativus														
Panax ginseng							200 mg	B					50 mg	B
L-arginine			2500 mg	C	60 mg	C			124 mg	C	114 mg	C		
Withania somnifera							300 mg	B						
Eurycoma longifolia														
Pinus pinaster														
Corynanthe yohimbe														
*L-taurine*					50 mg	D					100 mg	D		
*L-carnitine*			160 mg	D										
*L-citrulline*														
*Muira puama*					200 mg	D					200 mg	D	100 mg	D
*Trigonella foenum-graecum*														
*Ginkgo biloba*													30 mg	D
*Hawthorn berry*														
*Punica granatum*														
*Cuscuta chinensis*														
*Black Pepper*														
*Zingiber officinale*											20 mg	D		
*Turnera diffusa*					100 mg	D					200 mg	D	50 mg	D
*Vitamin C*									16 mg	D				
*Vitamin B6*	0.42 mg	D												
*Vitamin B9*									50 mcg	D				
*Vitamin E*														
*Vitamin D*														
*Vitamin B1*	0.33 mg	D												
*Vitamin H*	15 mcg	D												
*Vitamin B12*	0.75 mcg	D												
*Vitamin B3*			20 mg	D							48 mg	D		
*Magnesium*	112.5 mg	D							76 mg	D				
*Zinc*							10 mg	D	1.5 mg	D				
*Selenium*														
*Alpha lipoic acid*									10 mg	D				
*Aspartic acid*											86 mg	D		
*Bambusa arundinacea*														
*Epimedium acuminatum*											10 mg	D		
*Capsicum annuum*													375 mg	D
*Caffeine*													25 mg	D
*Paullinia capuana*														
*Chlorella pyrenoidosa*														
*Lepidium meyenii*					200 mg	D	300 mg	D			200 mg	D	100 mg	D
**Active Ingredient**	**DS 15**		**DS 16**		**DS 17**		**DS 18**		**DS 19**		**DS 20**		**DS 21**	
	**S = −0.20**		**S = −0.56**		**S = 0**		**S = 0.21**		**S = −0.38**		**S = 0.90**		**S = −1**	
	**Daily Dose**	**EV**	**Daily** **Dose**	**EV**	**Daily Dose**	**EV**	**Daily Dose**	**EV**	**Daily Dose**	**EV**	**Daily Dose**	**EV**	**Daily Dose**	**EV**
Tribulus terrestris	200 mg	C	108 mg	C	150 mg	C	200 mg	C			125 mg	C		
Crocus sativus														
Panax ginseng							150 mg	B	100 mg	B	80 mg	B		
L-arginine	500 mg	C	364 mg	C			240 mg	C	200 mg	C	125 mg	C		
Withania somnifera														
Eurycoma longifolia														
Pinus pinaster														
Corynanthe yohimbe														
*L-taurine*	200 mg	D	30 mg	D			200 mg	D						
*L-carnitine*														
*L-citrulline*			63.7 mg	D					600 mg	D				
*Muira puama*			30 mg	D							150 mg	D		
*Trigonella foenum-graecum*					300 mg	D								
*Ginkgo biloba*														
*Hawthorn berry*														
*Pomegranate*														
*Cuscuta chinensis*														
*Black Pepper*							30 mg	D	20 mg	D				
*Zingiber officinale*														
*Turnera diffusa*														
*Vitamin C*									100 mg	D				
*Vitamin B6*													10 mg	D
*Vitamin B9*													400 mcg	D
*Vitamin E*									24 mg	D				
*Vitamin D*													10 mcg	D
*Vitamin B1*														
*Vitamin H*														
*Vitamin B12*													5 mcg	D
*Vitamin B3*														
*Magnesium*													190 mg	D
*Zinc*	15 mg	D	7.5 mg	D									10 mg	D
*Selenium*													100 mcg	D
*Alpha lipoic acid*														
*Aspartic acid*													650 mg	D
*Bambusa arundinacea*														
*Epimedium acuminatum*			9 mg	D										
*Capsicum annuum*														
*Caffeine*			180 mg	D										
*Paullinia capuana*							180 mg	D						
*Chlorella pyrenoidosa*									100 mg	D				
*Lepidium meyenii*	500 mg	D	80 mg	D			200 mg	D	300 mg	D	220 mg	D	3000 mg	D
**Active Ingredient**			**DS 22**		**DS 23**		**DS 24**		**DS 25**		**DS 26**		**DS 27**	
			**S = 0.30**		**S = 2**		**S = 0.30**		**S = 2**		**S = 0.30**		**S = 1.33**	
			**Daily Dose**	**EV**	**Daily Dose**	**EV**	**Daily Dose**	**EV**	**Daily Dose**	**EV**	**Daily Dose**	**EV**	**Daily Dose**	**EV**
Tribulus terrestris			30 mg	C	525 mg	C	250 mg	C	2000 mg	B	250 mg	C		
Crocus sativus													14 mg	B
Panax ginseng			21 mg	B	150 mg	B							150 mg	B
L-arginine					1350 mg	C			40 mg	C			400 mg	C
Withania somnifera														
Eurycoma longifolia											50 mg	B		
Pinus pinaster					60 mg	B								
Corynanthe yohimbe							100 mg	B	200 mg	B				
*L-taurine*														
*L-carnitine*														
*L-citrulline*					84.6 mg	D							50 mg	D
*Muira puama*			200 mg	D										
*Trigonella foenum-graecum*														
*Ginkgo biloba*							120 mg	D						
*Hawthorn berry*														
*Punica granatum*														
*Cuscuta chinensis*														
*Black Pepper*														
*Zingiber officinale*														
*Turnera diffusa*														
*Vitamin C*														
*Vitamin B6*											3 mg	D		
*Vitamin B9*														
*Vitamin E*														
*Vitamin D*														
*Vitamin B1*														
*Vitamin H*														
*Vitamin B12*														
*Vitamin B3*														
*Magnesium*											140 mg	D		
*Zinc*			20 mg	D	7 mg	D					8 mg	D	14.7 mg	D
*Selenium*														
*Alpha lipoic acid*														
*Aspartic acid*														
*Bambusa arundinacea*														
*Epimedium acuminatum*							500 mg	D	500 mg	D				
*Capsicum annuum*														
*Caffeine*														
*Paullinia capuana*														
*Chlorella pyrenoidosa*														
*Ledidium meyenii*			50 mg	D			250 mg	D	600 mg	D			1180 mg	D

## Data Availability

Not applicable.
